# Retraction Note: Investigating the nexus among environmental pollution, economic growth, energy use, and foreign direct investment in 6 selected sub-Saharan African countries

**DOI:** 10.1007/s11356-024-32894-x

**Published:** 2024-03-15

**Authors:** Max William Ssali, Jianguo Du, Isaac Adjei Mensah, Duncan O. Hongo

**Affiliations:** 1 School of Management, Jiangsu University, Zhenjiang, 212013 People’s Republic of China; 2 Faculty of Science, Jiangsu University, Zhenjiang, 212013 People’s Republic of China; 3 School of Finance, Department of Statistics, Jiangsu University, Zhenjiang, 212013 People’s Republic of China

**Retraction Note: Environmental Science and Pollution Research (2019) 26:11245-11260**

10.1007/s11356-019-04455-0

The Publisher has retracted this article in agreement with the Editor-in-Chief. An investigation by the publisher found a number of articles, including this one, with a number of concerns, including but not limited to compromised peer review process, inappropriate or irrelevant references, containing nonstandard phrases or not being in scope of the journal. Based on the investigation's findings the publisher, in consultation with the Editor-in-Chief therefore no longer has confidence in the results and conclusions of this article.

The authors disagree with this retraction.

